# “Save your eyes for thirty Rupees”: A case study

**Published:** 2017

**Authors:** Asim Kumar Sil

**Affiliations:** Netra Niramay Niketan, Vivekananda Mission Ashram, Chaitanyapur, Purba Medinipur, West Bengal

**Figure:**
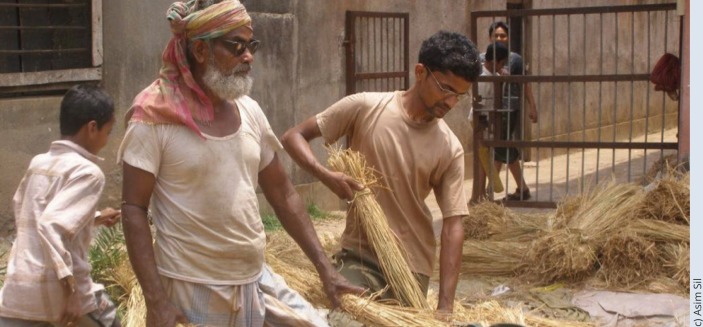
A farm labourer wears protective glasses during threshing. INDIA

**Figure:**
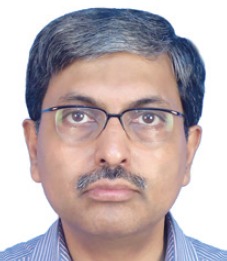
Asim Kumar Sil, DO, DNB, MSc

## Introduction

Various types of agricultural eye injuries are common in India. The prevalence of ocular injury in agricultural workers is unknown in India but data from a few studies suggest that this is quite common.^1^,^2^ Injury from sugarcane leaf is quite common in Northern and Western India, grape vine injury is common in central and south India.^3^ Paddy grain injury of cornea is very common in coastal India where rice is grown as the main crop. In recent times, the incidence of paddy grain injury has gone up because mechanical (paddle or power driven) threshers have replaced the traditional practice of manual separation of grains by beating the plant against a raised wooden platform.^4^ This article depicts the experience of a community-based intervention for preventing corneal injury in agricultural workers in a rural area of West Bengal.

## Paddy grain and the eye

Agriculture in West Bengal is the means of livelihood of about 65% population. Rice occupied almost 53% of the total agricultural crop of the state during 2007–2008.^4^

The age-old practice of separation of paddy grains from the plant used to be by hitting the tip of the plant against a platform made of bamboo or wood. This process takes longer time and involves more manpower. Since the speed with which the grain comes out during separation is less, there is less chance of eye injury by the paddy. Now the process has been replaced by the use of mechanical threshers to do the same work in much shorter time and save resources.^4^

In most areas of West Bengal rice is grown in 3 different seasons. They are called Aus (autumn rice), Aman (winter rice) and Boro (summer rice). The sowing time of summer rice is November to February and harvesting time is March to June. The average yield of Boro is 50 – 60% higher than other two varieties.^4^ Plants grown in different seasons are not of the same length, varying from 100 to 190 cm.^5^ In the Southern part of Bengal one high yielding variety of rice is harvested during April-May. This plant is short in length and has a greater number of grains than the other ones. That is why cases of corneal abrasion are much more reported during April-May. One study from South India has reported a higher incidence of fungal keratitis occurring during the months corresponding to the harvest seasons, during which time infection from vegetative corneal injury may be more likely.^6^

During harvesting almost the entire family of a farmer is involved in the work. Mechanical threshers are usually operated by young men by feet and the tip of the plant is placed over the spin. Another person, usually a woman constantly sweeps the ground to collect the grains at one place. Her face is usually closer to the machine and more prone to injury. Anybody, even a child moving close to the thresher may get injured.^7^

Farmers have a habit of covering the head and face with a piece of cloth to avoid dust but leave the eyes open while threshing. This practice keeps the eyes unprotected. The commonest mode of injury is abrasion of the cornea by rapidly moving seed. The most unfortunate sequel of this injury is development of fungal keratitis. Paddy grain has fine hair like structures over the outer coating, which is why the grains gets firmly anchored to the conjunctiva.

Sometimes the grain lodges inside the upper fornix and remains unnoticed, and in rare cases, it may start growing inside the eye.^7^ Treatment of fungal keratitis is difficult in rural locations, as the cases often report late and are complicated by the use of unknown eye drops or native medications. Most dangerous is the application of topical steroids which are sold over the counter in village medical shops without prescription, as steroids worsen fungal infections. Fungal culture facility is usually not available in rural situations and, antifungal medications if available are therefore used empirically.

Application of too many drops often reduces the efficacy of antibiotics. All these issues contribute to unilateral corneal blindness after paddy grain injury and often patients are of active working age. Morbidity, loss of time, work and ultimate of loss of vision make paddy grain injury a public health issue.

## Prevention of paddy injury – community intervention

The most obvious way of preventing this corneal injury is protecting the eyes at the time of threshing. Wearing plastic goggles was considered to be a cheap and easy option. Education materials were produced to propagate the use of protective glass. Posters were displayed in places that farmers visit usually. Eye health talks were organized in different occasions and festivals. One short public education video “only 30 Rupees to save your Vision,” was developed in local language to motivate people to wear glasses (plastic goggles in India cost Rupees 30 or half a Dollar). This video was shown in different places including the local cable network. A compact disc of this six minute film was distributed among volunteers who used it locally. This video is widely used during eye donation awareness meetings also.

The most effective way of communication was interactive meeting with the farmers. Farmers' co-operatives were selected for the meetings. Every large village in this part of Bengal has one co-operative where farmers get agricultural assistance and the evening is the suitable time to get them there. Interactive meetings started with the thought provoking video and was followed by discussions. It was found that many farmers do the threshing in the evening using electric lights. Sometimes it is overtime work, or to avoid daytime heat. Initially dark glasses (used after cataract surgery) were promoted to avoid corneal abrasion. But these were unsuitable for evening use, so the dark glasses were replaced with plain ones without increasing the cost. This white goggle had more acceptances, especially among women. The price could be kept under INR 30. The message that any kind of spectacle is good to protect the eyes was conveyed through this intensive approach.

## Measuring the impact

Sutahata and Mahishadal Blocks of East Medinipur district in West Bengal were selected for intensive campaigning few weeks before the harvesting time. These blocks were selected because of the proximity to the hospital. This is also the closest eye care facility for the villagers. The population of these blocks was approximately 356,000.

We looked at the hospital data of all cases of corneal ulcers from these two locations as well as from Purba and Paschim Medinipur districts served by our hospital. A decreasing trend is observed over time in those two selected blocks.

## Discussion

This awareness campaign could have made some impact in preventing corneal injury and reduction of corneal ulcer. There could be various reasons for reduction in number of walk in patients with corneal ulcers in the clinic. Awareness campaign is possibly one contributing factor.

There are always barriers in the usage of safety eyewear amongst workers. In one study from central India about three-fourths of the workers reported using it all or most of the time during work.^8^ Despite knowing that protective eyewear devices offer safety from work-related injuries, workers do not tend to use them for multiple reasons. These include some blurring of vision, discomfort, fogging, unusual appearance, people making fun of them, slipping of the goggles due to sweat and slowing work pace.

Prevention of ocular injuries in agriculture workers will reduce the incidence of microbial keratitis amongst them. Srinivasan et al demonstrated that treating corneal abrasions with antibiotic ointment by health workers at the village level signicantly reduced the incidence of bacterial and fungal corneal ulcers, but primary prevention of injury is always the best.^9^ It is all about developing the attitude of adopting safety measures. Constant effort of educating the community will result in consciousness about eye safety and develop peer pressure to wear protective goggles. Providing protective goggles at an affordable cost should complement this effort. Also, the manufacturers of the threshers have a responsibility in ensuring safety of the agricultural workers by modifying the design.

Awareness will always remain as the main strategy for prevention of eye injury. The current approach is interactive and participatory. The experience with a small defined population encourages us to scale up the campaign involving all stakeholders and making the goggles available locally.

## Acknowledgement

Dr. Samar K. Basak, Director, Disha Eye Hospitals, Barrackpore, West Bengal Ms. Barnali Banerji, Assistant Director of Agriculture, Directorate of Agriculture, Kolkata, West Bengal.

**Table 1.** Number of cases of corneal ulcers in areas where the community intervention was applied

Year	Sutahata Block	Mahishadal Block	Purba Medinipur District	Paschim Medinipur District
2009	48	60	687	244
2010	58	62	705	307
2011	69	69	703	312
2012	92	74	804	329
2013	126	58	852	336
2014	89	56	826	310
2015	46	48	875	360
